# Deep Learning-Based Link Quality Estimation for RIS-Assisted UAV-Enabled Wireless Communications System

**DOI:** 10.3390/s23198041

**Published:** 2023-09-23

**Authors:** Belayneh Abebe Tesfaw, Rong-Terng Juang, Li-Chia Tai, Hsin-Piao Lin, Getaneh Berie Tarekegn, Kabore Wendenda Nathanael

**Affiliations:** 1 Department of Electrical Engineering and Computer Science, National Taipei University of Technology, Taipei 10608, Taiwan; keskes2007@gmail.com; 2 Institute of Aerospace and System Engineering, National Taipei University of Technology, Taipei 10608, Taiwan; hplin@ntut.edu.tw; 3 Department of Electrical and Computer Engineering, National Yang Ming Chiao Tung University, Hsinchu 30010, Taiwan; j.tai@nycu.edu.tw (L.-C.T.); gechb21@gmail.com (G.B.T.); 4 Department of Electronic Engineering, National Taipei University of Technology, Taipei 10608, Taiwan; nooptanio2007@gmail.com

**Keywords:** link quality estimation, reconfigurable intelligent surfaces (RIS), gated recurrent unit (GRU), unmanned aerial vehicle (UAV)

## Abstract

In recent years, unmanned aerial vehicles (UAVs) have become a valuable platform for many applications, including communication networks. UAV-enabled wireless communication faces challenges in complex urban and dynamic environments. UAVs can suffer from power limitations and path losses caused by non-line-of-sight connections, which may hamper communication performance. To address these issues, reconfigurable intelligent surfaces (RIS) have been proposed as helpful technologies to enhance UAV communication networks. However, due to the high mobility of UAVs, complex channel environments, and dynamic RIS configurations, it is challenging to estimate the link quality of ground users. In this paper, we propose a link quality estimation model using a gated recurrent unit (GRU) to assess the link quality of ground users for a multi-user RIS-assisted UAV-enabled wireless communication system. Our proposed framework uses a time series of user channel data and RIS phase shift information to estimate the quality of the link for each ground user. The simulation results showed that the proposed GRU model can effectively and accurately estimate the link quality of ground users in the RIS-assisted UAV-enabled wireless communication network.

## 1. Introduction

In the future, wireless communication systems will face unprecedented demands for high-quality communication services, representing significant challenges for the current telecommunications industry [1]. Fortunately, unmanned aerial vehicles (UAVs) are being utilized as aerial wireless communication platforms to improve communication coverage and data rates [2]. These UAVs have the advantages of high deployment flexibility and dynamic mobility features. UAV-aided wireless communication systems are a promising component for future communication systems and could support a wide variety of applications with orders of magnitude more capacity than current systems [3]. In the future, UAVs will play a critical role in high-speed wireless communications, among many other applications enabled by UAVs [4]. By deploying UAVs with wireless communication capabilities, it will be possible to establish temporary wireless networks in areas where infrastructure is limited or damaged. This flexibility could also be useful in emergencies, such as natural disasters or search and rescue operations, in which the communication infrastructure is disrupted [4].

UAVs are used to support the current communication infrastructure in order to provide uninterrupted communication services in the designated area. UAVs, with their flexibility and low operating costs, have become a promising option for future wireless networks [5]. With their adjustable heights and mobile capabilities, UAVs can establish line-of-sight (LoS) links that provide reliable communication. However, UAVs’ power limitations and large path losses associated with long distances due to non-line-of-sight (NLoS) connections can make it difficult to ensure good communication services. Fortunately, recent advances in technology have led to the emergence of reconfigurable intelligent surfaces (RISs) as a promising solution for addressing the challenges faced by UAVs. By integrating passive reflecting elements with low-cost electronics, RIS technology is expected to improve electromagnetic propagation behavior and enhance communication quality. This technology is primarily driven by the increasing demand for 6G applications and future Internet of Things (IoT) networks [6]. RISs work by reflecting radio signals in a controlled manner, allowing them to reach their intended destination with higher quality and a longer range [7].

In recent years, there has been significant focus on using RIS to assist with wireless communications for aerial base stations (ABS) [8]. In the context of ABS wireless communication, RIS can be deployed on the ground or on the UAV itself to create a smart reflecting surface that can improve link quality and coverage. The RIS can reflect the wireless signals transmitted by the ABS to create multiple reflections that enhance signal strength and coverage. The RIS can also adaptively adjust the reflected signals to minimize interference and enhance signal quality [9]. Overall, RIS-assisted UAV wireless communication systems have the potential to significantly improve the communication performance of UAVs, particularly in challenging environments such as urban areas or remote locations with limited infrastructure [10].

UAV-enabled wireless communication link quality estimation (LQE) is intended to enhance the quality and reliability of ABS communication networks [11]. The accuracy of LQE directly impacts the system’s overall performance [12]. However, LQE in UAV systems is challenging due to the mobility and instability of UAVs, leading to time-varying and unpredictable channels. Moreover, the quality of wireless communication between the UAV and the ground users can be severely impacted by various factors, such as signal fading, interference, and path loss. Moreover, in an RIS-assisted UAV communication system, the UAV is constantly moving, and the channel conditions are constantly changing due to different factors [13]. Therefore, accurate and fast LQE is critical to enable efficient beamforming and reflection coefficient adaptation to maintain a reliable communication link [14]. Therefore, the motivation for RIS-assisted UAV wireless communication in LQE is to enhance the system’s performance by providing a stable and controllable environment, mitigating signal attenuation, and improving communication performance leading to accurate and reliable LQE.

According to the authors in [12], the LQE can be improved, leading to better communication performance and increased data transmission rates in UAV communication. The use of RIS for UAV LQE requires sophisticated algorithms to accurately estimate the link quality and determine the optimal configuration of the RIS elements. It is possible to create models using deep learning techniques that can predict the quality of the link based on the configuration of the RIS and the location and trajectory of the UAV.

In this paper, we proposed a deep learning-based model that estimates link quality in RIS-assisted UAV-enabled wireless communication systems for multiple ground users, taking into account the UAV’s trajectory and the RIS’s phase shift. To implement the proposed model, we employ a gated recurrent unit (GRU). The main contributions of our work can be summarized as follows:

(1) Numerous studies in the literature assess the quality of links in communication systems that utilize UAVs [11,12,13]. This article accurately estimates link quality in UAV-based communication systems by considering slow fading and fast fading while integrating RIS. We create a communication system for urban users integrating RIS and UAV. This system effectively addresses issues with signal propagation between UAVs and users in urban environments, accounting for building blockage effects.
(2) We propose a GRU-based model for estimating link quality in UAV-assisted wireless networks by leveraging the full capabilities of UAV and RIS, including considering the UAV trajectory and RIS passive phase shift.
(3) We provide numerical results that demonstrate the significance of the proposed model in terms of accuracy for estimating the link quality with the assistance of RIS in an RIS-assisted UAV communication system.

The remainder of our paper is organized into six sections. In Section 2, we describe the related work. Section 3 describes the system model. We provide a detailed explanation of our proposed method and GRU link quality estimation framework in Section 4. We present simulation results in Section 5 to evaluate our proposed solution’s performance. Lastly, Section 6 concludes the report with some final remarks.

## 2. Related Work

In [15,16,17,18,19], LQE was analyzed under different wireless network scenarios. Usually, authors study conventional wireless communication structures, but this paper examined future wireless communication scenarios, specifically RIS-assisted UAV-enabled wireless communication systems.

In [15], a stacked autoencoder-based link quality estimator (LQE-SAE) was proposed for a wireless sensor network in order to estimate the link quality effectively. The authors used the SAE model to extract the asymmetric characteristics of the downlink and uplink. To determine the link quality indicators, the authors used the received signal strength and signal-to-noise ratio. SAE fuses these characteristics to construct the link features’ vectors. These vectors are then input into the support vector classifier, which is labeled with the link quality grade. In [16], the authors used a support vector machine algorithm with training features available from the received signal strength indicator (RSSI) for conventional wireless communication systems. An evaluation was conducted to quantify the design decisions made during the development of a machine-learning-based LQE. The evaluation was completed using a publicly available dataset. In [17], the authors conducted aerial-to-ground link stability experiments and proposed a stochastic model to predict link quality, throughput, mission duration, and connection/disconnection durations in UAV and wireless sensor networks. In [18], the authors proposed a new method for estimating link quality in air-to-ground (A2G) wireless channels using long short-term memory (LSTM) in a 3D aerial base station deployment. This adaptive and accurate approach achieved the highest level of accuracy in LQE. In [19] the authors proposed a time-series convolutional neural network (CNN)-based link quality estimation model used to determine the link quality at each timeslot for drone-based wireless communication via amplitude and phase measurement data. The authors used the model results to deploy drone-based station wireless communication. 

The studies in [15,16,17,18], estimated link quality for conventional wireless communication, with some examining UAV-enabled wireless communication systems. Traditional learning approaches and shallow machine learning approaches have limitations in link quality estimation accuracy due to the proposed system scenario. However, our work focuses on an RIS-assisted UAV-enabled communication system rather than a UAV communications system. our proposed model uses deep learning approaches for link quality estimation by integrating RIS phase shift information into user channel data to estimate the link quality between the UAV and ground users. A wireless environment is also prone to multipath fading and attenuation of objects due to environmental factors. Therefore, we propose a data-driven deep learning technique that has powerful learning capabilities for estimating link quality with non-linear correlations in emerging advanced communication environments. This feature is vital for RIS-assisted UAV communication because it allows the system to optimize its communication performance by dynamically adjusting the RIS parameters. Accurately estimating link quality helps determine optimal UAV deployment and communication throughput. Thus, the goal of LQE for RIS-assisted UAV wireless communication is to estimate link quality accurately for the dynamic configuration of the RIS and select the best position of the UAV for the deployment of effective RIS-assisted UAV communication systems.

## 3. System Model

**Figure 1:**
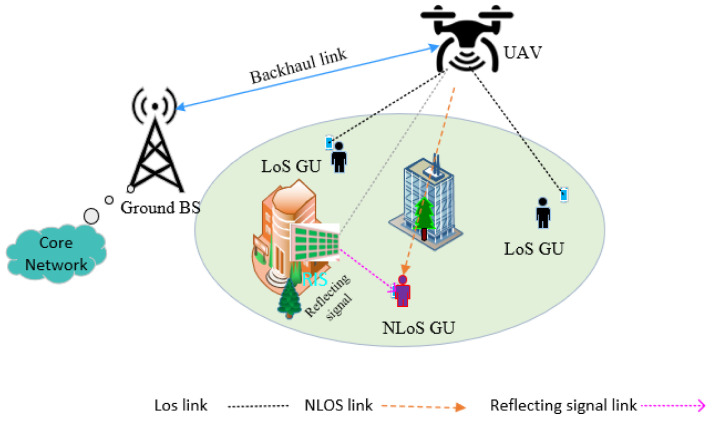
The RIS-assisted UAV-enabled wireless communications system.

In our work, we analyzed a downlink transmission system and omnidirectional antenna equipped on a UAV, multiple ground users, and an RIS located on a building. In this system, a single UAV *d* is deployed as an aerial base station to provide continuous wireless services for a set *U* = {1, 2 …, *u*} of *U* fixed ground users (GUs), as shown in Figure 1 of the RIS-assisted UAV wireless communication system. The UAV connects to the core network via a backhaul link. In practice, there is no direct link between the UAV and the GUs at any moment due to blockage by a building or tree. Thus, RIS is deployed on buildings to generate another virtual link for NLoS ground users. All the ground users are assumed to be equipped with a single antenna. The RIS are equipped with *N* reflecting elements, and with each set denoted as *N* = {1, …, *n*}. The location of each GU *u* and RIS is denoted by q^u^ = (*x_u_*, *y_u_*) and q^r^ = (*x_r_*, *y_r_*), respectively, but the location of the ground user and RIS are fixed. In addition, the UAV d can be located in space $L_{d}^{}=(x_{d}^{},y_{d}^{},h_{d}^{})$, where $\left(x_{d}^{},y_{d}^{}\right)$ is the latitude and longitude coordinates of the UAV d and $h_{d}^{}$ is the flight fixed altitude of UAV *d*. Changing the longitude and latitude coordinates allows the UAV to move on a horizontal trajectory.

### 3.1. Channel Model

In our system model, UAVs usually fly at high altitudes and RIS are commonly placed on the facade of a building. As shown in Figure 1, the system model communications channels are composed of the direct channel (i.e., the UAV-GU link) and the channel reflected through RIS that reflects the signals from the UAV (i.e., the UAV-RIS and the reflected signal’s RIS-GU link). In the RIS-assisted UAV communication system, we have two main links: the direct link (UAV-GUs link) and the reflecting link (UAV-RIS-GUs link).

#### 3.1.1. UAV-GU Link

The channel of the direct link between the UAV and the GUs is $h_{t}^{d,u}$ ∈ C ^1×1^. The most commonly used models of A2G communication apply simplified LoS channels. However, we use the statistical LoS channel (i.e., the probabilistic LoS channel) [20] and consider a more practical A2G channel model. This model is characterized by large-scale and small-scale fading calculated based on a simulated map considering the presence of buildings as propagation scatterers [21]. The channel between the UAV and GUs during timeslot *t* can be determined as follows:

(1) $$h_{t}^{d,u}=\sqrt{G_{t}^{d,u}}\Omega_{t}^{d,u}$$

where $G_{t}^{d,u}$ is large-scale fading or path loss and $\Omega_{t}^{d,u}$ is small-scale fading [4]. The large-scale fading coefficient can be written as reflected:

(2) $$G_{t}^{d,u}=10^{-p_{t}^{d,u}/10}$$

where $p_{t}^{d,u}$ is the path loss. The path loss between the UAV and the GUs can be written as follows:

(3) $$p_{t}^{d,u}=\left\{ \begin{array}{l} PL_{t}^{d,u}+\eta LoS,\;if\;LoS\;link\\ PL_{t}^{d,u},if\;NLoS\;link \end{array}\right.$$

where $PL_{t}^{d,u}=20\;log_{10}{\;D}_{t}^{d,u}+20\;log_{10}\;fc+20\;log_{10}\left(\;\frac{4\pi }{c}\right)$ denotes the FSPL (free space path loss), $D_{t}^{d,u}=∥L_{t\;}^{d}-q^{u}∥$ denotes the distance between the UAV and the u-th GUs, and ηLoS denotes the propagation loss of the LoS links, *fc* is the carrier frequency and *c* is the speed of light.

Small-scale fading $\Omega_{t}^{d,u}$ is modeled via Rician fading with factor K_1_, which can be written as follows [5]:

(4) $$\Omega_{t}^{d,u}=\left(\sqrt{\frac{K_{1}}{K_{1}+1}}{\tilde{h}}_{t}^{d,u}LoS+\sqrt{\frac{1}{K_{1}+1}}{\tilde{h}}_{t}^{d,u}NLoS\;\right)$$

where K_1_ is the Rician factor that quantifies the relative strength of the LoS component compared to the scattered components in the channel. ${\tilde{h}}_{t}^{d,u}LoS$ denotes the deterministic LoS component with |${\tilde{h}}_{t}^{d,u}LoS$| = 1 and ${\tilde{h}}_{t}^{d,u}NLoS$ is a random NLoS component.

#### 3.1.2. UAV-RIS-GU Link

If the direct link (UAV-GU link) is blocked, scatters are still generated through RIS. The reflecting link (UAV-RIS-GU link) at time *t* consists of two LoS sub-links: the UAV-RIS sub-link and the RIS-GU sub-link. The indirect link from the UAV to the GU includes the channel between the UAV and the RIS, which is denoted by $\;h_{t}^{d,r}$ ∈ C^1×^^*N*^, and the channel between the RIS and the GU, which is denoted by $h_{t}^{r,u}$ ∈ C*^N^*^×1^. The channel gain between the UAV and the RIS at timeslot *t* is as follows:

(5) $$h_{t}^{d,r}=\sqrt{\frac{\alpha }{{\left(D_{t}^{d,r}\right)}^{2}}}\left[1,e^{-j\frac{2\Pi }{\lambda }\dot{d}\phi_{t}^{d,r},\ldots \ldots ,-j\frac{2\Pi }{\lambda }(N-1)\dot{d}\phi_{t}^{d,r}}\right]$$

where α represents the path loss at a distance of 1 m, which serves as the reference point, and $D_{t}^{d,r}=∥q_{t\;}^{d}-q^{r}∥$ denotes the distance, $\phi_{t}^{d,r}=\frac{x_{t}^{d}-x^{r}}{D_{t}^{d,r}}$ and represents the cosine of the angle of departure of the signal at timeslot *t* [5]. The number of reflecting elements is denoted as *N.*

For channel gain between the RIS and u-th GUs at the *t*-th timeslot, the antenna separation distance is $\dot{d}$, and the carrier wavelength is *λ*.

(6) $$h_{t}^{r,u}=\sqrt{\frac{\alpha }{{\left(D_{t}^{r,u}\right)}^{\beta }}}\left(\;\sqrt{\frac{K1}{K1+1}}\;{\left[1,e^{-j\frac{2\Pi }{\lambda }\dot{d}\phi_{t}^{r,u},\ldots \ldots ,-j\frac{2\Pi }{\lambda }(N-1)\dot{d}\phi_{t}^{r,u}}\right]}^{T}+\sqrt{\frac{1}{K1+1}}{\tilde{h}}_{t}^{r,u}NLoS\;\right)$$

where α represents the path loss at a distance of 1 m, which serves as the reference point, β is the RIS-ground user link phase loss exponent, $D_{t}^{r,u}=∥q^{r}-q^{u}∥$ represents the distance from the RIS to the GU, $\phi_{t}^{r,u}=\frac{x_{t}^{r}-x^{u}}{D_{t}^{r,u}}$ represents the cosine of the angle of arrival of the signal, *N* is the RIS number of the reflecting element, $\dot{d}$ is the antenna separation distance, *λ* is the carrier wavelength, and ${\tilde{h}}_{t}^{r,u}NLoS$ is random NLoS component [5].

Consequently, the total of the two sub-links is $h_{t}^{dru}$ for the UAV-RIS-GU link. The blocked user can access this link via the virtual link with the help of RIS. The UAV-RIS-GU link can be written as:

(7) $$h_{t}^{dru}=h_{t}^{d,r}\Theta_{t}^{}h_{t}^{r,u}$$

(8) $$\Theta_{t}^{}=diag\left(\theta_{t}^{n}\right)\;\in \;C^{N\times N}$$

(9) $$\theta_{t}^{n}=\left[\vartheta_{t}^{1}e^{j\phi_{t}^{1}}\;,\ldots ,\vartheta_{t}^{n}e^{j\phi_{t}^{n}}\right]{\;}^{T}\;\in \;C^{N\times 1}$$

where $\Theta_{t}^{}\;$ is the RIS phase shift matrix, $\theta_{t}^{n}$ is the reflection coefficient, $\vartheta_{t}^{n}$ ∈ [0, 1] is the amplitude and $\phi_{t}^{n}$ ∈ [0, 2π] is the phase shift of the subsurface *n* of RIS at time slot *t* [22].

In RIS, the phase shift is a critical parameter that determines how the RIS elements modify the phase of the incident wave. The RIS phase shift introduced by each RIS element can be classified into two categories: discrete phase shifts and continuous phase shifts [23]. Both types of phase shifts are essential for manipulating and optimizing the propagation of electromagnetic waves in various wireless communication scenarios [15]. In this paper, we select a discrete phase shift due to its simplicity in implementation and ability to reduce complexity. To set a discrete phase shift value, we first determine the number of bits or levels of phase resolution. Thus, we choose a 4-bit phase resolution that will produce 16 (2^4^) discrete phase shift values. Based on the range of phase shift values the RIS elements can take 0 to 2π degrees. In this case, the phase values are spaced at 22.5^0^ with a range from 0 to 2π degrees.

At time step *t*, the signal received by the user device on the ground at the *u*-th is written as:

(10) $$y_{t}=(h_{t}^{d,u}+h_{t}^{dru})\sqrt{p_{o}}x+\alpha^{2}$$

where α^2^ is the noise signal following the complex Gaussian distribution *CN* (0, α^2^), *x* is the signal and *P_o_* is the UAV transmission power.

Then, based on (1) and (7), the received signal-noise ratio (SNR) of the GUs at the n-th time slot can be written as:

(11) $$SNR=\frac{{\left|h_{t}^{d,u}+h_{t}^{dru}\right|}^{2}p}{No}$$

where *P* is the UAV transmission power, and *N_o_* is the UAV noise power.

The level of link quality for the user is assessed using the link quality indicator (LQI), which takes into account the SNR. The SNR parameter is indeed a commonly used metric for assessing link quality in various communication systems, including those used in UAVs [24]. In the context of UAV communication, a higher SNR generally indicates a stronger and more reliable signal, which entails better link quality. A high SNR ensures that the received signal is less susceptible to noise and interference, resulting in improved data transmission rates, reduced errors, and increased overall system performance. When designing and optimizing UAV communication systems, monitoring and maintaining a sufficient SNR level is crucial for ensuring reliable and efficient data transmission. The SNR is typically measured at the receiver and can be used as a link quality indicator for assessing the performance of the communication link. Therefore, it is important to use the SNR along with other relevant metrics to evaluate the overall link quality in UAV communication systems.

## 4. Proposed Method

### 4.1. GRU-Based Link Quality Estimation Model

In this work, we proposed a GRU algorithm to build a LQE model. The GRU is a type of recurrent neural network (RNN) shown to be effective in capturing sequential dependencies in data [25]. The GRU is particularly useful when dealing with sequential data of varying lengths, making it suitable for modeling time-varying link quality. Depending on the task complexity and the amount of training data available, the specific architecture and configuration of the GRU layers must be designed. Using historical channel data, the model predicts the desired output (link quality indicator). GRU is more accurate and computationally efficient than LSTM when working with large datasets [25]. Therefore, we proposed a GRU-based LQE framework to learn spatial-temporal features from channel data efficiently. 

**Figure 2:**
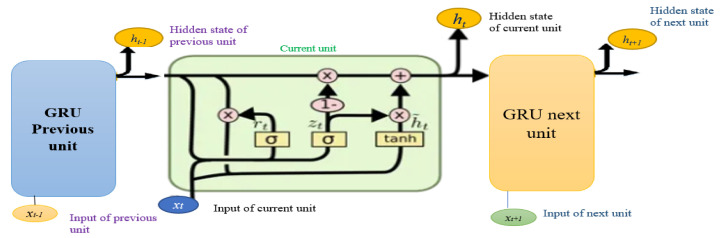
GRU memory blocks.

Figure 2 depicts the architecture of the GRU network. The first box represents the previous unit information, the middle box denotes the current unit, and the third box signifies the future unit information. The manipulation of the current unit relies on the performance of the gates, the current inputs, and the hidden layer. Within this architecture, the update gate determines whether to transmit the preceding information from the previous unit (h_t−1_) to the current or subsequent unit (h_t_). The hidden layer’s current state at a given time is updated by linearly interpolating between h_t−1_ and the current state h_t_. The reset gate merges new input with stored data, while the update gate determines information utilization. Without an output gate, the GRU can be considered a distinct approach for integrating and combining information. The GRU network has a simplified structure compared with LSTM [25]. The connection relationship in Figure 2 gives the following equations:

(12) $$r_{t}=\sigma (W_{xr}^{T}.x_{t}+W_{hr}^{T}.h_{t-1}+b_{r})$$

(13) $$zt=\sigma (W_{xz}^{T}.xt+W_{hz}^{T}.h_{t-1}+b_{z})$$

(14) $${\bar{h}}_{t}=\tanh(W_{x\bar{h}}^{T}.xt+W_{h\bar{h}}^{T}.(rt⊗h_{t-1})+b_{\bar{h}})$$

(15) $$h_{t}=zt⊗h_{t-1}+(1-z_{t})⊗{\bar{h}}_{t}$$

In a GRU, *x* is the input vector, *h* is the output vector and *Wxr*, *Wxz*, and *Wx* are the weight matrices for the input vector. $\bar{h}$ is the candidate output vector, and *W_hr_*, *W_hz_*, and *W_h_* are the weight matrices for the previous time step. Additionally, *b_r_*, *b_z_*, and *b*$\bar{h}$ are the bias terms. The element-wise multiplication operation is represented by ⊗. σ and tanh represent the logistic sigmoid and hyperbolic tangent functions, respectively. The GRU has two gates: *r* is the reset gate and z is the update gate.

In a GRU network, each GRU unit includes a reset gate (*r*) and an update gate (*z*) that handles past, present, and future data [26]. The update gate (*z*) controls the history and newly added channel data with the RIS phase shift, which can be computed according to (12). Meanwhile, the reset gate (*r*) controls the flow of new input data to the preceding GRU unit and can be computed according to (13). To calculate the current hidden unit, two steps are involved. Firstly, a vector of new candidate values updates the cell state through (14). Secondly, the candidate hidden unit takes two input vectors the current input vector (*r_t_*) and the previous hidden unit vector (*h_t−_*_1_) and multiplies them by the gr output. The result then passes through the tanh activation function to determine the candidate hidden unit. Finally, the current hidden unit (*h_t_*) is calculated using (15) [27].

**Figure 3:**
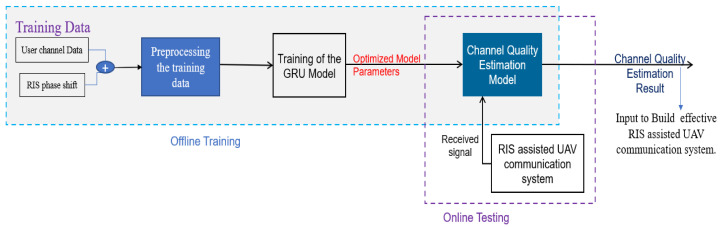
The Architecture of the proposed link quality estimation system.

To leverage the effectiveness of a GRU model for LQE in RIS-assisted UAV-based wireless communication, we prepared user channel simulation data and preprocessed simulated data, trained the GRU model on these data, and then used the trained model to produce estimations and decisions about the communication parameters to optimize link quality during UAV operations. This process can help ensure reliable and high-performance wireless communication in dynamic and challenging environments. The proposed LQE architecture is classified into three stages: data collection, preprocessing of the training dataset, and building the LQE model, as shown in Figure 3.

#### 4.1.1. User Channel Data Simulation

We used the MATLAB R2023a simulation tool to collect user channel data and RIS phase shift information. Through the simulation tool, we created a simulation environment containing a UAV, RIS, and users. After creating the simulation environment, we established the simulation environment to set up the parameters. After we defined the parameters, we generated the channel data according to the channel model mathematical expression in Section 3.1. In simulating the RIS phase shift we defined our used RIS parameters such as the number of elements and reflection coefficients. We calculate the RIS phase shift depending on the channel modeling and desired signal properties. In the next time slot, we update the phase shift at each RIS element based on the channel conditions and the specific algorithms being used. Finally, we generate user channel data and the RIS phase shift by analyzing the received signal and extracting the relevant information for each user. This paper used a UAV with a fixed altitude and a horizontal trajectory; thus, at each UAV trajectory, we collect simulation user channel data. The RIS phase shift algorithm uses 9 elements of RIS to obtain data at a single UAV location for a single user. We obtained the channel gain from each UAV location with each RIS element and from each user via two direct links (UAV-GU) and virtual links (UAV-RIS-GU).

We collected data from each position of the UAV by changing the nine RIS elements. To express the mathematical expressions, we represent each position of the UAV with the index variable ‘j’, ranging from 1 to d, and represent each RIS element with the index variable ‘k’, ranging from 1 to 9. Based on these notations we express the given statements as a mathematical expression:

Data collected = ∑∑D_s_ (j, k)

where, D_s_ (j, k) represents the data collected at UAV position “j” and RIS element “k”. Here, the inner summation (Σ) runs over the RIS elements in each position (k = 1 to 9). Hence, the above expression assumes that the data collected at each UAV position and RIS element can be combined in a meaningful way. Based on these data, we trained the model.

In this paper, we use the RIS phase shift algorithm. Algorithm 1 summarizes how the RIS phase shift is performed. Algorithm 2 summarizes the process of performing the RIS phase shift. The process starts by initializing the RIS phase shift value as 0 and setting the maximum number of phase shift iterations depending on the number of RIS. The algorithm then enters a loop where it obtains the current CSI from the base station, calculates the optimal phase shift value based on the CSI, updates the RIS phase shift value, and increases the iteration counter by one. This loop continues until the iteration counter reaches the maximum number of iterations. Finally, the algorithm ends. By following this algorithm, the RIS phase shift can be effectively performed, allowing for improved wireless communication performance in RIS-assisted UAV communication systems.
**Algorithm 1:** RIS Phase Shift Algorithm.  **Input:** RIS parameter value   **Output:** Final configuration of the RIS elements  1:  $\mathrm{Initialize}\;\mathrm{the}\;\theta_{t}^{n}$ = 0  2:  **For** RIS element: = 1 to N **do**        // N is elements of RIS  3:      $\mathrm{Compute}\;\theta_{t}^{n}$ using Equation (9)  // compute the reflection coefficient  4:    Compute $\phi_{t}^{n}$  5:    Update $\theta_{t}^{n}$  6:  **end for**

The user data channel and RIS phase information can be denoted in the matrix as

(16) $$X=\left[\begin{array}{c} r_{1}\\ r_{2}\\ .\\ .\\ r_{I} \end{array}\right]$$

where *X ∈ X^MxF^ M* denotes the total records of the data, F is the number of features r_i_ is a row vector in the *i*th row.

#### 4.1.2. Data Preprocessing

The data include channel data and RIS phase shifts. After acquiring the data from the target area, pre-processing is carried out to develop an accurate LQE model. Data pre-processing consists of filling in missing values, data labeling (i.e., SNR to LQI), and data normalization to improve the training process and enhance neural networks’ performance. We fill in the missing value preprocessing techniques in UAV communication for users who do not receive signals as this issue produces missing values. A user who does not receive a signal may be given a noise value, indicating that −6 should be filled in that missing SNR value after which we apply data labeling to the data. Next, by filling in missing values, we label different link quality levels based on the SNR value. For example, y = 1 for LQI = poor (SNR, less than 10), y = 2 for LQI = Fair (SNR, 10–20), y = 3 for LQI = good (SNR, 20–30), y = 4 for LQI = very good (SNR, 30–40), and y = 5 for LQI = excellent (SNR, greater than 40). Then, we use the min-max normalization technique [17]. The link quality parameters in UAV communication are different. The data are normalized between 0 and 1 to eliminate the range and reduce model error. The min–max normalization technique enables the data to be independent of the range and reduces the model errors. This measure also helps the model learn more from the data and facilitates further analysis of the data. For this process, we applied the min–max normalization formula, which maps the data between 0 and 1. This process allows us to compare data from different ranges and make more accurate predictions. Furthermore, the min–max normalization technique ensures that the data are not affected by outliers, making the model more robust. This technique also helps reduce the time needed for training the model. The normalization process is outlined in (17) as follows:

(17) $$s^{\prime }=\frac{\left(s-min\left(s\right)\right)}{\left(max\left(s\right)-min\left(s\right)\right)}$$

where s is the original data and s’ is the normalized data. After normalization, the data are ready to be fed into the neural network. Normalization helps the neural network to learn faster and more accurately and prevents the network from becoming stuck in local minima. Finally, this process ensures that the weights of the network are not skewed by the presence of outliers in the data.
**Algorithm 2:** Preprocessing data.  **Input:** User channel data and RIS phase shift  **Output:** Return filled missing values, labeled data, and normalized values. // **Step 1**. Filling Missing Values:  1:  **For** future of dataset: =f_d_ to F_d_    // f_d_ is future of da-taset   2:    **For** sample: = s_d_ to S_d_   // s_d_ is sample of dataset   3:      **If** f_d_ is missing:   4:        Replace the missing value by −6   5:       **end if**
  6:    **end for**
  7:  **end for** // **Step 2**. Data Labeling:   1: **For** sample: = s_d_ to S_d_   2:    Label the SNR to LQI based on the above range   3:    Assign the corresponding label to the sample   4:  **end for** // **Step 3**. Data Normalization:   1:  **For** the future of the dataset: =f_d_ to F_d_   2:      **For** sample: = s_d_ to S_d_   3:        Normalize the value of the feature using the (16)   4:       **end for**   5:  **end for**

Algorithm 2 provides a comprehensive approach for preprocessing user channel data with an RIS phase shift. By filling in missing values, labeling data, and applying min-max normalization, the resulting data are made ready for further analysis and modeling tasks.

#### 4.1.3. Build Link Quality Estimation Model

To address the LQE problem of RIS-assisted UAV communication systems, we employ a GRU algorithm. Here, simulated user channel data and the RIS phase shift information are used as input. Among the various channel data parameters, we consider the SNR to label the link quality indicator for the GRU-based LQE model. The GRU model is then trained on the user channel data and RIS phase shift dataset. Once the features are normalized as described in the preprocessing section using Algorithm 2, the GRU model is applied. Accordingly, GRU-based LQE model training uses a normalized dataset as inputs and the corresponding LQIs as outputs. During training, the trial-and-error method is used to adjust the network hyperparameters [28] until the optimal GRU model is achieved. As part of the RIS-assisted UAV communication system, a well-trained LQE model is uploaded to the UAV to continuously monitor the link quality. Algorithms 3 and 4 outline the detailed training process for the GRU LQE Model.
**Algorithm 3:** GRU-based LQE Method.   **Input:** preprocessed dataset.   **Output:** Optimized w and b for training the LQE model.    // w is weight, b is bias  1: Initialize the learning parameters of GRU.  2: For epoch: =1 to T do  3:      LQE model Training;  4:      GRU network weights and bias need to be updated;  5: **end for**

LQE model offline training: We use the input data X = (*r_1_*, *r_2_*,…, *r_I_*) to denote the sequence of input data vectors and Y for the corresponding LQI. The proposed LQE model is trained using preprocessed user channel data and the corresponding LQI. Next, the model predicts the output’s LQI.
**Algorithm 4:** Online LQE model.       1: load the Algorithm 3 model:      2:  **For** each TS t do       3:    received SNR from user;       4:      estimate link quality       5:        t = t + 1;       6:  **end for**

For the LQE model online training, after being trained offline, the LQE model is used by the UAV to predict ground user link quality over time with Algorithm 4. The estimated ground user link quality is input to optimize UAV mobility for the deployment of RIS-assisted UAV-enabled wireless communications.

## 5. Results

### 5.1. Simulation Setup

In this scenario, a single UAV transmits a signal to GUs and an RIS is deployed on a building within a 200 m × 200 m urban area. The RIS is located at the coordinates [40, 74, 50]. L_d0_ = [27, 121, 50] is the UAV’s initial location and flies 50 m at a fixed altitude. The timeslot length is set to 1 s and the number of timeslots is set to 250. MATLAB R2023a is used to conduct the user channel data simulations. The simulation parameters are listed in Table 1.

### 5.2. Results and Discussion

We conducted experiments collecting RIS phase information and user channel data; 80% of the data were selected to train the LQE model while the remaining 20% were used for testing. To prepare the data for the model, Algorithm 1 preprocessed the data into a suitable structure. 

**Figure 4:**
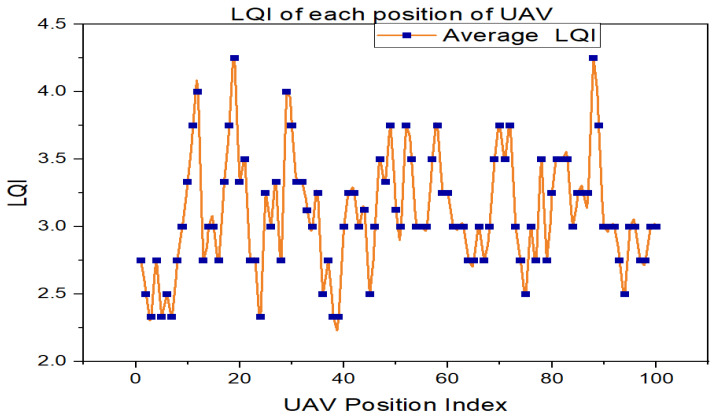
Average LQI value performance with respect to each position of UAV.

The performance of each position of the UAV in terms of the LQI value can be analyzed to identify which UAV position is most suitable to serve the GUs. In this paper, we consider 100 UAV positions. Thus, we must analyze each position of the LQI value to identify the best UAV position and link. Figure 4 shows the performance of each UAV position in terms of the average LQI value.

As shown in Figure 4, positions 12, 19, 29, and 88 have the highest LQI values, which means that these positions’ UAVs have better communication performance or superior links with ground users; thus, we can deploy the UAVs directly in the corresponding positions. This information can help optimize UAV trajectory planning, resource allocation, and communication protocols to enhance overall network performance. However, when considering mobility ground users, we need to develop optimized UAV trajectory control and phase shift optimization to provide better communication service in the target area. Accordingly, our proposed LQE model helps to accurately deploy RIS-assisted UAV-enabled communication systems to provide continuous wireless service.

In an RIS-assisted UAV communication system, deploying an RIS near the UAV’s communication path can enhance the link quality between the UAV and the ground station or other UAVs. Each element of an RIS can reflect incident signals with different phase shifts. In our scenario, we used a 3 by 3 matrix (3 × 3) or nine elements of RIS, each element is active during all time slots but each element of RIS that reflects performance depends on the locations of the UAV and ground user.

**Figure 5:**
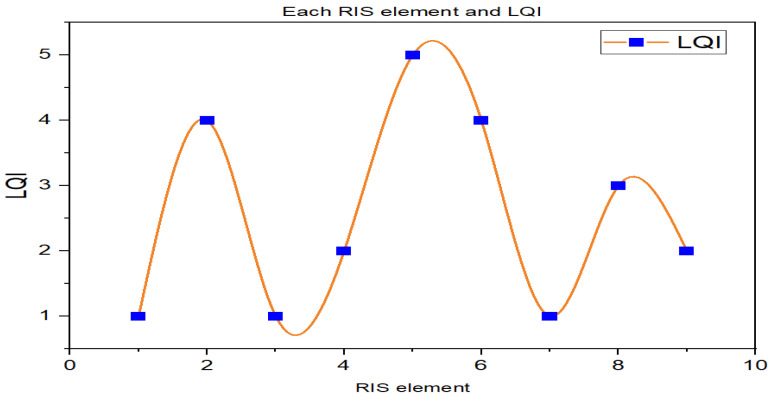
The LQI performance of ground users in each element of RIS.

Figure 5 shows the average LQI performance of each RIS element. According to the results of the experiments from all RIS elements, the fifth element has a higher LQI on average than the other elements of RIS. Thus, in an RIS-assisted communication system, by measuring all elements of the RIS, we can select the best element of the RIS to achieve superior signal complexity, as well as the best link between the UAV and the ground users. We can improve the overall performance of the communication links by selecting the best element of the RIS. This process can be used to improve the connection between UAVs and ground users.

**Figure 6:**
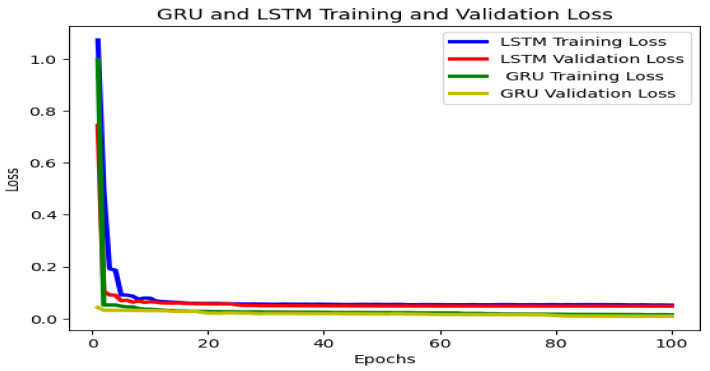
Performance losses of the LSTM and GRU LQE model.

To build the proposed GRU system we consider a preprocessed dataset. Additionally, we compare the GRU and the LSTM algorithms using common parameters, evaluating the GRU algorithm’s performance. The evaluation was conducted using similar layers, optimizers, batch sizes, and epochs to avoid bias. Table 2 lists the optimal learning hyperparameters for GRU-based link quality estimation. Figure 6 shows a comparison of the link quality estimation performance of GRU and LSTM. As the training and validation epochs increase in size, the fitting lines diverge in the LSTM algorithm. The GRU model, on the other hand, indicates better performance on larger datasets with fewer overfitting issues. Thus, GRU is the best option for avoiding bias in system performance evaluations. The GRU model is, therefore, also preferred for use in larger datasets, as this model is better equipped to handle high complexity and high-volume data while maintaining accuracy. This makes the GRU model the optimal choice for many deep-learning applications.

In Figure 6, both LSTM and GRU exhibit fluctuations in the original data as the number of epochs increases, but both algorithms show improved performance. However, the GRU algorithm presents a greater performance loss of over 0.058. Additionally, the data suggest that GRU offers better integrity than the other algorithms for accurate link quality predictions.

We used accuracy and cross-entropy to evaluate the model, as shown in Table 3. Here, the LSTM and GRU models predict cross-entropy values of 0.12 and 0.062, respectively, the original data. The GRU offers a better cross-entropy value than LSTM, indicating that this method is better for the link quality estimation algorithm in RIS-assisted UAV communication systems.

We developed the GRU LQE model based on the hyperparameters in Table 2 to estimate LQI. Making predictions with newly available data can be used to estimate link quality for unseen samples using the trained GRU model. 

**Figure 7:**
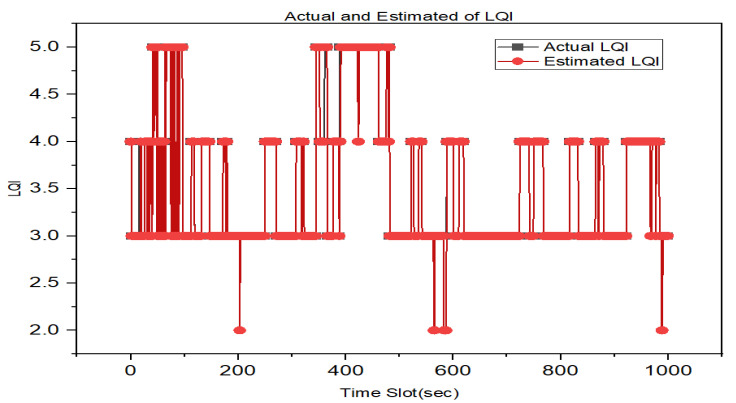
Prediction performance of GRU LQE model.

As shown in Figure 7, the GRU model correctly predicted the LQI. The link quality value is accurately estimated when a system achieves optimal performance.

We also undertook a comparison between performance with and without RIS link quality estimations. Specifically, based on our developed model we compared the link quality between the UAV and ground users by considering UAV communications with and without an RIS.

**Figure 8:**
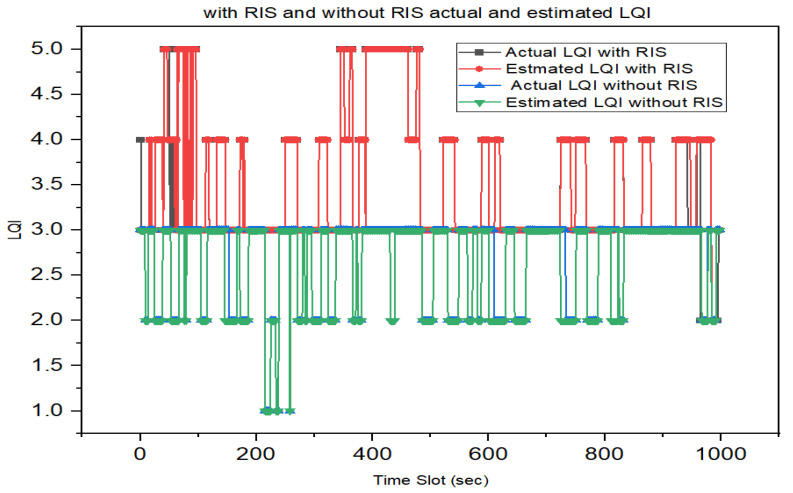
The GRU LQE estimation performance of RIS-assisted and without RIS-assisted UAV communication.

Figure 8 shows a comparison between performance with and without RIS link quality estimations. According to the experiment, with RIS the average link quality is 3–4 LQI values, which means that the system offers excellent quality communication. In contrast, without RIS the average link quality is 1–3 LQI, which indicates poor to good quality communication. Thus, with RIS the UAV communication system is more powerful and capable of better communication than without RIS UAV communication system. Therefore, RIS-assisted UAV communication systems offer significant improvements in link quality compared to traditional UAV communication without RIS. RIS technology allows for dynamic adaptation to changing environments, interference mitigation, and focused signal transmission. These processes lead to enhanced reliability, coverage, and link quality in UAV communications. However, the specific benefits of RIS will depend on the optimization algorithms, number of RIS elements, deployment scenarios, and other system parameters.

### 5.3. Computational Time and Complexity

The LQE model requires less computation time and complexity to assess the quality of communication links at each time slot. To reduce the computation time and complexity of the LQE model, we selected the GRU algorithm for our proposed system because the GRU algorithm model architecture has fewer parameters and computations, resulting in faster training and inference. Additionally, the training time of GRUs often converges faster during training due to their simpler gate mechanisms of GRUs. This simplicity can lead to shorter training times and also reduce memory consumption. GRUs typically require less memory because they have fewer parameters; this can be advantageous when working with limited computational resources. In terms of theoretical time complexity, GRU has O(t) complexity per time step, where ‘t’ is the sequence length. Therefore, GRUs often have a computational advantage due to their simpler architecture and fewer parameters, leading to faster training and limited computational resources. Hence, we implemented and optimized the GRU-based link quality estimation model by optimizing the hyperparameters, reducing unnecessary computations, and ensuring efficient memory usage. Moreover, we applied data preprocessing techniques to achieve faster convergence during training and more efficient estimation.

## 6. Conclusions

In this paper, we proposed a link quality estimation model for RIS-assisted UAV-enabled communications systems to improve the performance of UAV-assisted wire-less communication systems with the assistance of RIS. RIS has been proposed as a helpful tool to enhance UAV communication networks. To estimate the link quality of a RIS-assisted UAV communication system, we proposed a GRU-based link quality estimation model that was developed to properly estimate the A2G wireless communication link. We evaluated our proposed system using simulated user channel data. The simulation results showed that utilizing GRU can effectively enhance the performance of link quality estimation for RIS-assisted UAV-enabled wireless communication with the assistance of RIS. In future work, we plan to focus on the deployment of RIS-assisted UAV wireless communication systems in a target area by considering 3D UAV trajectory and RIS phase shift in a dynamic environment to significantly improve channel fading effects, as well as further enhance user data rate and communication coverage.

## Figures and Tables

**Table 1:** Simulation Parameter.

Parameter	Value
Transmit power p	0.1 W
Noise Power No	−80 dB
Path loss at 1 m α	−20 dB
Path loss for LoS ηLoS	0.2 dB
Path loss for NLoS ηNLoS	21 dB
Carrier frequency fc	2 GHz
Rician factor K1	3 dB
Speed of light c	3 × 10^8^ m/s
Path loss RIS-GU link β	2.8
Number of RIS elements N	9
quantization bits b	4 bits

**Table 2:** The deep learning network parameters.

Parameter	Value
Number of hidden layers	3 (128, 64, 32 neurons)
Dropout	0.2
Batch size	32
Learning rate	0.001
Loss function	Cross-entropy
Optimization algorithm	Adam
Number of epochs	100

**Table 3:** LQE performance evaluation.

Performance Metrics	LSTM	GRU
Accuracy	0.95	0.96
Cross-entropy	0.12	0.062

## Data Availability

Not applicable.
